# A descriptive study of reportable gastrointestinal illnesses in Ontario, Canada, from 2007 to 2009

**DOI:** 10.1186/1471-2458-12-970

**Published:** 2012-11-12

**Authors:** Linda Vrbova, Karen Johnson, Yvonne Whitfield, Dean Middleton

**Affiliations:** 1 Canadian Field Epidemiology Program, Public Health Agency of Canada, 120 Colonnade Rd, Ottawa, ON, Canada; 2 Public Health Ontario, 480 University Avenue, 3rd floor, Toronto, ON, Canada; 3 School of Population and Public Health, University of British Columbia, 2206 East Mall, Vancouver, British Columbia, Canada; 4 Dalla Lana School of Public Health, University of Toronto, 155 College St., Health Sciences Building, 6th floor, Toronto, ON, Canada

## Abstract

**Background:**

Gastrointestinal illnesses (GI) continue to pose a substantial burden in terms of morbidity and economic impact in Canada. We describe the epidemiology of reportable GI in Ontario by characterizing the incidence of each reportable GI, as well as associated demographics, clinical outcomes, seasonality, risk settings, and likely sources of infection.

**Methods:**

Reports on laboratory confirmed cases of amebiasis, botulism, campylobacteriosis, cryptosporidiosis, cyclosporiasis, giardiasis, hepatitis A, listeriosis, paratyphoid fever, salmonellosis, shigellosis, typhoid fever, illness due to verotoxin-producing *Escherichia coli* (VTEC-illness), and yersiniosis, from January 1, 2007 to December 31, 2009 were obtained from Ontario’s passive reportable disease surveillance system. Cases were classified by history of relevant travel, association with outbreaks, and likely source of infection, obtained through follow-up of reported cases by local health authorities.

**Results:**

There were 29,897 GI reported by health authorities in Ontario from 2007 to 2009. The most frequently reported diseases were campylobacteriosis (10,916 cases or 36.5% of all GI illnesses) and salmonellosis (7,514 cases, 25.1%). Overall, 26.9% of GI cases reported travel outside of Ontario during the relevant incubation period. Children four years of age and younger had the highest incidence rate for most GI, and significantly more (54.8%, p<0.001) cases occurred among males than females. The most commonly reported sources of infections were food (54.2%), animals (19.8%), and contact with ill persons (16.9%). Private homes (45.5%) and food premises (29.7%) were the most commonly reported exposure settings. Domestic cases of campylobacteriosis, cryptosporidiosis, giardiasis, salmonellosis, and VTEC-illness showed seasonal patterns with incidence peaking in the summer months.

**Conclusions:**

Reportable GI continues to be a burden in Ontario. Since more than one in four GI cases experienced in Ontario were acquired outside of the province, international travel is an important risk factor for most GI. Because private homes are the most commonly reported risk settings and the main suspect sources of infection are food, animal contact and ill persons, these findings support the continued need for public health food safety programs, public education on safe handling of food and animals, and proper hand hygiene practices.

## Background

Gastrointestinal illnesses (GI) continue to be an important global public health issue. While mortality from GI is decreasing, particularly in children in developing countries, morbidity worldwide remains high [1]. In Canada, the incidence of GI is estimated to be 1.3 episodes of acute GI per person-year, and the probability that an individual develops GI in a year is estimated to be 72% [2]. The economic costs of each case are substantial: the mean annual cost associated with GI is estimated to be Can$115 per capita and Can$1,089 per case [3].

In Ontario, Canada’s most populous province with 12.9 million inhabitants in 2008 [4], there are 15 pathogen-specific GI that are reportable to 36 local public health authorities, who, in turn, report centrally to the province. Previous epidemiological analyses of GI in Ontario describe cases reported from 1997 to 2003 [5-7]. The purpose of this study is to present the epidemiology of reportable GI in Ontario from 2007 to 2009. Specifically, we report on the incidence of each reportable GI, their demographics, outcomes, seasonality, likely sources of infection and exposure settings, with the goal of informing the development and evaluation of prevention and control policies and programs.

## Methods

### Data sources

De-identified case reports for this study were provided by the Ontario Ministry of Health and Long-Term Care (MOHLTC) in accordance with a data sharing agreement between the MOHLTC and Public Health Ontario (formerly known as the Ontario Agency for Health Protection and Promotion). The integrated Public Health Information System (iPHIS) is Ontario’s central reportable disease surveillance system and is the source of case data for this study. The data used in this study are not posted publicly; however, they are available through a data request to the MOHLTC. Confirmed case reports were provided for 15 pathogen-specific GI with associated episode dates (earliest of onset date, specimen collection date, or reported date) from January 1, 2007 to December 31, 2009. Case records for amebiasis, botulism, campylobacteriosis, cryptosporidiosis, cyclosporiasis, giardiasis, hepatitis A, listeriosis, paratyphoid fever, salmonellosis, shigellosis, typhoid fever, illness due to verotoxin-producing *Escherichia coli* (VTEC-illness), and yersiniosis were provided. Case definitions for all of these diseases are available online [8]. Due to small numbers, cholera was excluded from this analysis. For the calculation of incidence rates, Statistics Canada population data for the province of Ontario by age, sex, and year were used.

### Demographics and outcomes

Cases were stratified by age and sex at time of illness. Age was categorized into seven age groups: under 1 year, 1–4 years, 5–14, 15–24, 25–44, 45–64, and 65 years and older. Cases were classified as hospitalized if a hospitalization date was reported; hospitalization status was coded as unknown (instead of not hospitalized) if no hospitalization date was reported. Similarly with deaths, cases with death dates were classified as deceased and all others were classified as unknown.

### Exposures

Local health authorities in Ontario follow up on cases of reportable GI to identify exposures during the incubation period of interest in accordance with the *Health Protection and Promotion Act*, Regulation 569. There are currently no standard follow-up forms or set timelines for initial case contact or follow-up for GI in Ontario; each exposure reported by a case and deemed relevant by the investigator is recorded in iPHIS. Because these exposures are assigned by public health professionals who may have additional information including food testing results and food premise investigation records, local public health authorities use this information to establish links between cases. These exposures can be used to perform source attribution, being similar to “expert elicitation” source attribution methods [9]. Details pertaining to the exposure date, location, setting, transmission mode and source were used to determine the primary exposure source and setting. Cases identified as “lost to follow-up”, “pending”, and “untraceable”, as well as those without exposure details were assumed to have not been successfully followed up by public health, and were coded as missing in specific analyses. Cases with multiple exposures were excluded from the source attribution analyses, except for cases with two exposures where one was reported as “unknown”. In these instances, the “unknown” exposure was removed and the remaining exposure was used to determine primary exposure source and setting. Cases that could not be coded into exposure categories because of limited or conflicting exposure details were labelled unclassifiable and excluded from analyses.

Primary exposure source was categorized as: 1) *unknown*, if the only exposure reported was “unknown”, 2) *travel*, if out-of-province travel was recorded in any of the exposure fields (travel information is to be entered into iPHIS only if the investigator deems it relevant, i.e. travel occurred within the incubation period), 3) *water* for drinking water (e.g. contaminated/untreated well or surface water) or recreational water exposures (e.g. swimming/bathing in a pool, hot tub, lake or river), 4) *person* for person-to-person transmissions, including breast-feeding, sexual and in-utero transmission, 5) *food*, if a food item was mentioned in any of the exposure fields, 6) *animals* for contact with live animals and animal products excluding food, and 7) *other*.

Primary exposure setting was categorized as: unknown (as noted above), out of province for travel cases, private home, food premises (e.g. restaurant, grocery store, bakery, deli, caterer, mobile food premise), and other (e.g. institutions, hospitals, long-term care homes, farms, schools, and day nurseries) to reflect the setting in which the reported exposure occurred.

### Case classifications

Cases were stratified based on travel history and association with a reported outbreak. Outbreak-related cases were those linked to a reported outbreak in iPHIS. Sporadic cases were defined as those not linked to an outbreak; however, the first case in each outbreak was also included as a sporadic case for analyses of exposures and seasonality. Cases that were followed up by public health were defined as domestic if no relevant travel history outside of Ontario was reported and as travel-related if a relevant history of travel outside of Ontario was reported.

### Descriptive analyses

Overall and age-specific incidences were calculated for each disease in addition to proportions of cases hospitalized, deceased, travel- and outbreak-related. Seasonal trends, stratified by travel status, were also determined. Due to the small number of domestic cases, botulism, typhoid fever and paratyphoid fever were excluded from the age-specific analyses, and botulism was excluded from the seasonality analyses. Proportions of males for each disease were compared to 0.5 (equal proportions) using the binomial distribution if the number of cases was ≤ 1,000, and the normal distribution when the number of cases was >1,000. The magnitude of missing data was examined through the quantification of excluded cases that were not successfully followed up by public health, as well as cases with unknown, unclassifiable or multiple exposures. All data coding and analyses were conducted using SPSS version 19 (SPSS Inc., an IBM Company). Figures were constructed using Microsoft Excel (version 2010).

## Results

### Overall description of reported cases

**Figure 1:**
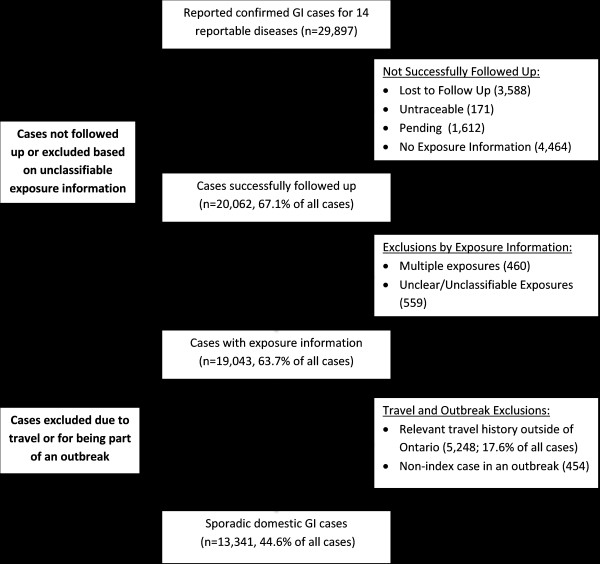
**Flow diagram of status of reported gastrointestinal illnesses in Ontario between 2007 and 2009**.

From 2007 to 2009, 29,897 gastrointestinal illnesses (GI) were reported by 36 local health authorities in Ontario. Of these, 20,062 (67.1%) were successfully followed up by public health. Figure 1 is a flow diagram of the various exclusions for all GI cases. Table 1 shows the proportion of cases not followed up by public health, as well as cases with multiple or unclear exposure information by disease. Amebiasis (54.3%), giardiasis (39.4%), and campylobacteriosis (39.1%) had the highest proportion of cases which were not successfully followed up.

**Table 1:** Proportion of gastrointestinal illnesses excluded from source attribution analyses

**Reportable disease**	**All reported cases: exclusions (%)**				
	**N**	**Not followed up**	**Multiple exposures**	No primary source**	**No primary setting†**
Amebiasis	2,134	54.3%	0.1%	0.7%	1.4%
Botulism	10	10.0%	0.0%	0.0%	10.0%
Campylobacteriosis	10,916	39.1%	1.2%	2.4%	4.9%
Cryptosporidiosis	1,048	20.9%	1.7%	3.6%	5.8%
Cyclosporiasis	341	15.8%	1.8%	1.5%	2.1%
Giardiasis	4,726	39.4%	0.6%	2.8%	3.8%
Hepatitis A	354	15.0%	2.8%	0.6%	4.2%
Listeriosis	189	19.6%	17.5%	3.2%	3.7%
Paratyphoid Fever	147	3.4%	0.0%	3.4%	0.0%
Salmonellosis	7,514	23.1%	2.0%	0.7%	3.9%
Shigellosis	727	13.1%	1.8%	1.0%	1.4%
Typhoid fever	259	3.9%	0.4%	0.0%	0.0%
VTEC-illness	760	17.4%	7.2%	4.7%	7.6%
Yersiniosis	772	27.1%	1.4%	0.6%	2.5%
***Total***	**29,897**	**32.9%**	**1.5%**	**1.9%**	**4.1%**

** Unable to classify the most likely source (based on case follow-up).

† Unable to classify the most likely setting where exposure took place (based on case follow-up).

Table 2 shows the number of reported cases and incidence by year. The most frequently reported GI were campylobacteriosis (10,916), salmonellosis (7,514) and giardiasis (4,726), which together accounted for 61.6% of all GI reported from 2007 to 2009. The proportion of cases attributed to travel and outbreaks, as well as the proportion of hospitalizations and deaths is shown in Table 3. Overall, 27.6% of GI cases were travel-related, having travelled outside of Ontario at any time during the relevant incubation period. The diseases with the highest proportion of cases related to travel were typhoid fever (87.1%), paratyphoid fever (85.1%), and cyclosporiasis (61.6%); the diseases with the lowest proportion of travel-related cases were botulism (0.0%), listeriosis (4.4%), and VTEC-illness (7.8%).

**Table 2:** Reportable gastrointestinal illnesses in Ontario by year, between 2007 and 2009

**Reportable disease**	**2007**		**2008**		**2009**		**Total**	
	**N**^**1**^	**Incidence /100,000**^**2**^	**N**^**1**^	**Incidence /100,000**^**2**^	**N**^**1**^	**Incidence /100,000**^**2**^	**N**^**3**^	**Mean annual incidence /100,000**^**4**^
Amebiasis	814	6.4	761	5.9	559	4.3	2,134	5.5
Botulism	*NP*	*NP*	*NP*	*NP*	*NP*	*NP*	10	0.0
Campylobacteriosis	3,883	30.3	3,789	29.3	3,244	24.8	10,916	28.1
Cryptosporidiosis	406	3.2	335	2.6	307	2.3	1,048	2.7
Cyclosporiasis	95	0.7	103	0.8	143	1.1	341	0.9
Giardiasis	1,612	12.6	1,610	12.4	1,504	11.5	4,726	12.2
Hepatitis A	120	0.9	114	0.9	120	0.9	354	0.9
Listeriosis	39	0.3	95	0.7	55	0.4	189	0.5
Paratyphoid Fever	46	0.4	58	0.4	43	0.3	147	0.4
Salmonellosis	2,819	22.0	2,385	18.4	2,310	17.7	7,514	19.4
Shigellosis	234	1.8	239	1.8	254	1.9	727	1.9
Typhoid Fever	86	0.7	96	0.7	77	0.6	259	0.7
VTEC-illness	317	2.5	278	2.1	165	1.3	760	2.0
Yersiniosis	269	2.1	260	2.0	243	1.9	772	2.0
*Total*	*10,746*	*84.0*	*10,125*	*78.3*	*9,026*	*69.1*	*29,897*	*77.1*

1 - N: The total number of cases reported annually (including domestic, travel, and unknown).

2 – N (per year)/population of Ontario the same year.

3 – N: The number of cases reported over the three years (including domestic, travel, and unknown).

4 - (N (all three years)/3)/mean population of Ontario over the three years.

NP - incidence not presented - incidence for Botulism <5 cases on average per year.

**Table 3:** Reportable gastrointestinal illnesses in Ontario by travel, outbreak, hospitalization, and mortality, between 2007 and 2009

**Reportable disease**	**% Travel-related**^**1**^	**Outbreaks**^**2**^**(n)**	**% Outbreak-related**^**3**^	**% Hospitalized**^**4**^	**% Deceased**^**5**^
Amebiasis	38.6% (31.9%-41.3%)	0	0.0% (0.0%-0.0%)	0.6% (0.4%-1.1%)	0.1% (0.0%-0.3%)
Botulism	0.0% (0.0%-0.0%)	0	0.0% (0.0%-0.0%)	60.0% (33.3%-100%)	0.0% (0.0%-0.0%)
Campylobacteriosis	20.4% (18.7%-21.2%)	10	0.7% (0.0%-1.1%)	3.4% (3.1%-3.8%)	0.1% (0.1%-0.2%)
Cryptosporidiosis	36.4% (35.7%-36.3%)	4	2.9% (0.0%-6.2%)	2.6% (2.2%-2.9%)	0.2% (0.0%-0.3%)
Cyclosporiasis	61.6% (59.6%-64.2%)	1	2.1% (0.0%-4.9%)	1.2% (1.0%-1.4%)	0.0% (0.0%-0.0%)
Giardiasis	39.0% (38.7%-39.4%)	5	0.4% (0.0%-0.9%)	1.0% (0.7%-1.2%)	0.2% (0.1%-0.4%)
Hepatitis A	46.7% (42.9%-50.6%)	2	1.4% (0.0%-2.5%)		20.9% (15.8%-25.4%)	0.6% (0.0%-1.7%)
Listeriosis	4.4% (3.4%-5.4%)	1	37.6% (0.0%-74.7%)	60.8% (48.7%-69.5%)	24.3% (16.4%-30.5%)	
Paratyphoid Fever	85.1% (82.5%-90.0%)	0	0.0% (0.0%-0.0%)	22.4% (17.4%-27.9%)	0.0% (0.0%-0.0%)	
Salmonellosis	21.3% (19.6%-23.1%)	32	6.1% (2.5%-11.4%)	6.9% (5.7%-7.9%)	0.6% (0.4%-0.7%)	
Shigellosis	48.4% (48.0%-48.7%)	2	1.5% (0.0%-4.7%)	8.0% (6.7%-9.8%)	0.1% (0.0%-0.4%)	
Typhoid Fever	87.1% (83.1%-92.6%)	0	0.0% (0.0%-0.0%)	44.4% (41.6%-48.8%)	0.0% (0.0%-0.0%)	
VTEC-illness	7.8% (4.8%-13.8%)	18	22.6% (6.1%-34.2%)	21.4% (19.6%-23.0%)	0.4% (0.0%-1.8%)	
Yersiniosis	18.3% (13.8%-24.7%)	0	0.0% (0.0%-0.0%)	4.0% (1.5%-5.8%)	0.1% (0.0%-0.4%)	
*Total*	*27.6% (26.6%-28.1%)*	*75*	*2.8% (1.1%-4.4%)*	*5.3% (5.0%-5.7%)*	*0.4% (0.3%-0.5%)*	

1 - The number of cases identified as travel-related, out of all cases with a defined travel status (i.e. excludes cases that were not followed up or could not be classified).

2 - The number of outbreaks.

3 - The number of outbreak related cases out of all cases, including travel, domestic and unknown (not followed up).

4 - The number of cases identified as being hospitalized out of the total number of reported cases.

5 - The number of cases identified as deceased out of the total number of reported cases.

NP - incidence not presented - incidence for Botulism low, <5 cases on average per year.

During this period, 75 outbreaks were reported. While these outbreaks were mostly due to salmonellosis (n=32 outbreaks, 460 cases), VTEC-illness (n=18 outbreaks, 172 cases) and campylobacteriosis (n=10 outbreaks, 72 cases), the highest proportion of outbreak-related cases were for listeriosis (37.6%, 71 cases) and VTEC-illness (22.6%, 172 cases). Hospitalization of more than 40% of reported cases was documented for listeriosis (60.8%), botulism (60.0%), and typhoid fever (44.4%). While the overall proportion of deaths for all GI combined was 0.4% (n=126), 24.3% (n= 46) of reported listeriosis cases died.

### Demographics of reported cases

**Figure 2:**
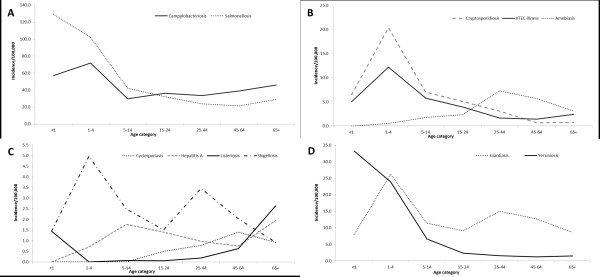
**Incidence of reportable gastrointestinal illnesses in Ontario by age group between 2007 and 2009 (N=29,449).A**: campylobacteriosis (n=10,901) and salmonellosis (n=7,507); **B**: amebiasis (n=2,134), cryptosporidiosis (n=1,046), verotoxigenic *Escherichia coli* (VTEC) illness (n=759); **C**: cyclosporiasis (n=341), hepatitis A (n=354), listeriosis (n=189), and shigellosis (n=726); **D**: giardiasis (n=4,721) and yersiniosis (n=771).

Figure 2 shows the age-specific incidence grouped by diseases with similar incidence levels. The highest age-specific incidence for salmonellosis and yersiniosis was in those under 1 year of age (Figure 2-A and 2-D). The highest incidence for campylobacteriosis, cryptosporidiosis, VTEC-illness, shigellosis, and giardiasis was in those aged 1–4, with shigellosis and giardiasis exhibiting a second, albeit lower peak in the 25–44 age group (Figure 2). Amebiasis and cyclosporiasis peaked in adults aged 25–44 and 45–64, respectively (Figure 2-B and 2-C). Listeriosis incidence was highest in those over 65 years of age, as well as in those under one year of age (Figure 2-C). The highest age-specific incidence for hepatitis A was in the 5–14 year age group (Figure 2-C).

Overall, significantly more GI cases occurred among males than females (54.8%, p<0.001). Males accounted for significantly more cases of amebiasis (71.1%, p<0.001), campylobacteriosis (55.0%, p<0.001), giardiasis (58.8%, p<0.001), shigellosis (57.2%, p<0.001), and yersiniosis (54.4%, p=0.02). There were significantly fewer male VTEC-illness cases (45.3%, p=0.01) and no significant sex differences were found for botulism, cryptosporidiosis, cyclosporiasis, hepatitis A, listeriosis, paratyphoid fever, salmonellosis, and typhoid fever.

### Exposures of domestic sporadic cases

Domestic sporadic cases that were successfully followed up by public health accounted for 44.6% (13,341/29,897) of all reported cases, and 70.1% (13,341/19,043) of all cases with exposures (Figure 1). Overall, 26.0% of primary exposure sources and 15.4% of primary exposure settings for domestic sporadic cases were known, with VTEC-illness having the highest proportion of known sources (40.7%) and settings (27.3%) of all diseases (Table 4).

**Table 4:** Sporadic domestic reportable gastrointestinal illnesses in Ontario by known or unknown exposure between 2007 and 2009

**Reportable disease**	**Domestic sporadic cases**	Primary source (%)**		**Primary setting (%)†**	
	**n**	**Known**	**Unknown**	**Known**	**Unknown**
Amebiasis	594	20.2%	79.8%	2.5%	97.5%
Botulism	9	11.1%	88.9%	11.1%	88.9%
Campylobacteriosis	4,982	25.5%	74.5%	16.0%	84.0%
Cryptosporidiosis	473	34.0%	66.0%	19.5%	80.5%
Cyclosporiasis	103	17.5%	82.5%	9.7%	90.3%
Giardiasis	1,646	20.9%	79.1%	10.0%	90.0%
Hepatitis A	152	35.5%	64.5%	10.5%	89.5%
Listeriosis	79	15.2%	84.8%	11.4%	88.6%
Paratyphoid Fever	21	9.5%	90.5%	4.8%	95.2%
Salmonellosis	4,107	28.0%	72.0%	18.4%	81.6%
Shigellosis	310	29.0%	71.0%	9.0%	91.0%
Typhoid Fever	32	6.2%	93.8%	6.2%	93.8%
VTEC-illness	388	40.7%	59.3%	27.3%	72.7%
Yersiniosis	445	18.9%	81.1%	14.6%	85.4%
***Total***	***13,341***	***26.0%***	***74.0%***	***15.4%***	***84.6%***

** The most likely source as identified on case follow-up (excludes cases with multiple exposures, travel cases, and outbreak cases).

† The most likely type of location where exposure took place based on case follow-up (excludes cases with multiple exposures, travel cases, and outbreak cases).

Among cases with known exposures, the most common sources were food (54.2%), animals (19.8%), and contact with an ill person (16.9%). Private homes (45.5%) and food premises (29.7%) were the most common exposure settings (Table 5). Among cases exposed in private homes (n=937), the most frequently reported sources were food (62.6%), followed by contact with animals (21.1%) and contact with persons ill with similar symptoms (13.1%). For cases exposed in food premises (n=611), the most frequently reported source was food (96.1%). Among cases exposed in settings other than private homes and food premises (n=510), the top exposure sources were contact with animals (38.0%), water (28.8%), and food (26.7%).

**Table 5:** Sporadic domestic reportable gastrointestinal illnesses in Ontario by exposure source and setting between 2007 and 2009

**Reportable disease**	Primary source (%)*						**Primary setting (%)†**			
	**n**	**Animal**	**Food**	**Person**	**Water**	**Other**	**n**	**Food premises**	**Private home**	**Other**
Amebiasis	*120*	*1.7%*	*8.3%*	*82.5%*	*5.8%*	*1.7%*	*15*	*33.3%*	*26.7%*	*40.0%*
Botulism	*1*	*0.0%*	*100.0%*	*0.0%*	*0.0%*	*0.0%*	*0*	*0.0%*	*0.0%*	*0.0%*
Campylobacteriosis	*1,272*	*26.9%*	*63.1%*	*6.0%*	*2.8%*	*1.3%*	*796*	*31.2%*	*47.5%*	*21.3%*
Cryptosporidiosis	*161*	*47.2%*	*9.3%*	*11.8%*	*29.2%*	*2.5%*	*92*	*2.2%*	*16.3%*	*81.5%*
Cyclosporiasis	*18*	*0.0%*	*100.0%*	*0.0%*	*0.0%*	*0.0%*	*10*	*70.0%*	*20.0%*	*10.0%*
Giardiasis	*344*	*14.2%*	*5.5%*	*36.0%*	*40.7%*	*3.5%*	*164*	*3.7%*	*33.5%*	*62.8%*
Hepatitis A	*54*	*0.0%*	*7.4%*	*87.0%*	*0.0%*	*5.6%*	*16*	*25.0%*	*50.0%*	*25.0%*
Listeriosis	*12*	*8.3%*	*75.0%*	*8.3%*	*8.3%*	*0.0%*	9	22.2%	44.4%	33.3%
Paratyphoid Fever	*2*	*0.0%*	*50.0%*	*50.0%*	*0.0%*	*0.0%*	1	0.0%	100.0%	0.0%
Salmonellosis	*1,148*	*15.2%*	*73.1%*	*9.9%*	*1.3%*	*0.4%*	754	37.7%	48.4%	14.0%
Shigellosis	*90*	*0.0%*	*16.7%*	*80.0%*	*0.0%*	*3.3%*	28	46.4%	50.0%	3.6%
Typhoid Fever	*2*	*0.0%*	*0.0%*	*50.0%*	*0.0%*	*50.0%*	2	0.0%	100.0%	0.0%
VTEC-illness	*158*	*18.4%*	*49.4%*	*19.6%*	*10.8%*	*1.9%*	106	28.3%	43.4%	28.3%
Yersiniosis	*84*	*15.5%*	*81.0%*	*1.2%*	*0.0%*	*2.4%*	65	15.4%	66.2%	18.5%
***Total***	***3,466***	***19.8%***	***54.2%***	***16.9%***	***7.6%***	***1.5%***	***2,058***	***29.7%***	***45.5%***	***24.8%***

* Primary source identified on case interview, excludes cases with multiple exposures, travel cases, outbreak cases, and cases with unknown exposures; “other” primary sources include fomites (clothing, towels), and environmental factors.

† Primary setting is the type of location where exposure took place based on case interview, excludes cases with multiple exposures, travel cases, outbreak cases, and cases with unknown exposures; “other” primary settings include hospitals, long-term care homes, prisons, schools, and campsite.

### Seasonality of domestic sporadic and travel cases

**Figure 3:**
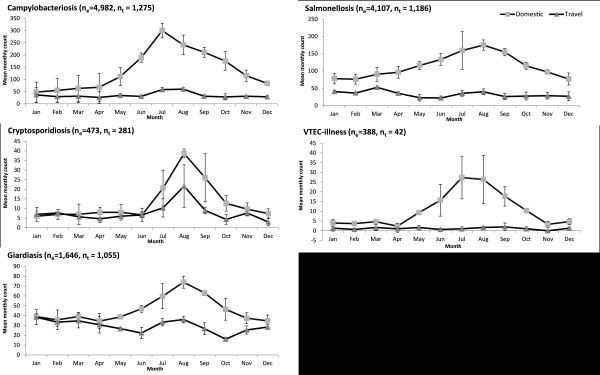
**Domestic and travel-related gastrointestinal illnesses in Ontario with pronounced domestic seasonal patterns.** Mean monthly number of sporadic domestic and travel-related reportable GI in Ontario with pronounced domestic seasonal patterns by travel status from 2007 to 2009. n_e_: number of sporadic domestic cases; n_t_: number of travel-related cases.

**Figure 4:**
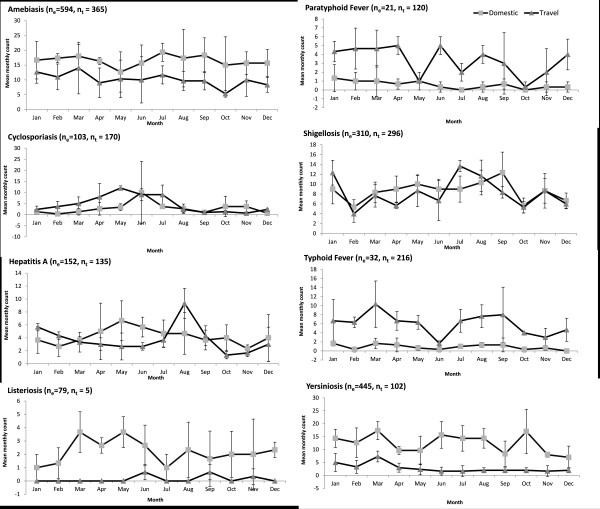
**Domestic and travel-related gastrointestinal illnesses in Ontario with many travel-related cases or without pronounced domestic seasonality.** Mean monthly number of sporadic domestic and travel-related reportable GI in Ontario with a high proportion of travel-related cases with or without pronounced domestic seasonal patterns by travel status for reported cases from 2007 to 2009. n_e_: number of sporadic domestic cases; n_t_: number of travel-related cases.

Domestic cases of campylobacteriosis, cryptosporidiosis, giardiasis, salmonellosis, and VTEC-illness showed seasonal patterns with incidence peaking in the summer months (Figure 3). For all these diseases, modest concurrent summer increases were also observed among travel-related cases with the exception of VTEC-illness. Additional peaks in travel-related cases in the winter for campylobacteriosis and giardiasis, and in early spring for salmonellosis, amebiasis, typhoid fever and yersiniosis were also observed (Figures 3 and 4). Cyclosporiasis was the only disease that showed a late spring/early summer peak in travel-related cases. Summer to early fall peaks were apparent in travel-related cases of shigellosis, hepatitis A and typhoid fever. Smaller peaks in January were seen in travel-related cases for most diseases (Figures 3 and 4).

## Discussion

### Incidence

Ontario documented annual incidence rates of reportable GI cases ranging from 69.2 to 83.9 per 100,000 persons per year for the years 2007 to 2009. Previous studies in Ontario reported on the same diseases, with the exception of the four parasitic diseases (amebiasis, cryptosporidiosis, cyclosporiasis, and giardiasis) included in the current study. As a result, direct comparisons to earlier studies are not easily made. Combined, these four parasitic diseases contributed 2,927, 2,809, and 2,513 cases in 2007, 2008, and 2009, respectively. If these diseases are excluded, the adjusted GI rates would be 61.1, 56.6, and 49.8 per 100,000 persons per year. For the years 1997 to 2003, the annual incidence rate ranged from a high of 89.8 in 1998 to a low of 60.2 per 100,000 persons in 2003 [5,7]. These rates suggest a slight decrease in the rate of GI in Ontario compared to a decade earlier.

Campylobacteriosis was the most frequently reported GI from 2007 to 2009, making up 36.5% of the total number of cases. The mean annual incidence rate of 28.1 per 100,000 persons for this period was lower than the 42.6 per 100,000 persons reported for the period 1997 to 2001 [7]. Salmonellosis was the second most frequently reported GI from 2007 to 2009, making up 25.1% of the total number of cases. The mean annual incidence rate of 19.4 per 100,000 persons for this period was slightly lower than the 22.6 per 100,000 persons reported for the period 1997 to 2001 [7]. It should be noted that since our study is based on reportable disease data, we could not include viruses that cause GI including the noroviruses, which are estimated to be responsible for 58% of GI caused by known pathogens in the United States, followed by nontyphoidal *Salmonella* spp. at 11% [10].

### Outbreaks

Listeriosis and VTEC-illness had the highest percentage of outbreak-related cases. The wide range in the annual proportion of cases associated with these two diseases was due to significant outbreaks of VTEC-illness in 2007 and 2008, and a large listeriosis outbreak in 2008.

### Demographics

Similar to previous studies, we found that children four years of age and younger have the highest incidence rate for most GI [5-7,11]. Overall, we found significantly more GI cases in males, also consistent with previous studies [5,6,11].

### Seasonality

There were pronounced seasonal trends in many of the diseases examined. All of the diseases exhibiting pronounced domestic seasonal patterns in this study increased in the summer; a pattern seen in previous Ontario studies [5-7,11]. This increase is thought to be due to increased outdoor activities (e.g. recreational water use), social gatherings with food (e.g. barbeques, picnics), an increase in mechanical vectors such as flies, and warmer temperatures that promote pathogen growth [5-7].

### Travel

Approximately one-quarter of GI cases were acquired outside of the province, a proportion similar to the 24.6% of cases related to travel between 1997 and 2001 [7]. Travel-related seasonal patterns were generally not the same as domestic seasonal patterns. Taylor et al. [12] also found that international travel had significant impact on the epidemiology of GI in the Canadian province of British Columbia, with the proportion of travel-related cases exceeding that of domestic cases for a group of 13 GI between January and March. This highlights the importance of completing exposure histories so that trends and rates for domestic and travel-related diseases can be assessed separately in order to properly target public health actions [12].

### Source attribution and risk setting

A wide diversity of bacterial, parasitic and viral infections cause GI, each with their specific complex transmission dynamics; GI pathogens can be transmitted from person to person, via contaminated food, water, fomites, as well as through direct or indirect contact with animals. There are a number of different approaches (microbiological, epidemiological, intervention studies and expert elicitation) used to attribute illnesses to specific sources, primarily for “foodborne illnesses”, where sources include animal reservoirs and vehicles (e.g. foods) [9].

In our study, we used the public health investigator’s determination of most likely setting and source of illness for each case, an approach used in all of the previous Ontario studies [5-7,11]. This approach to source attribution was also used by Dumoulin et al. [13] in a region of Ontario, who successfully used standardized questionnaires to determine the most likely source of infection from public health interviews. While the level of evidence for this type of source attribution is considered to be low, we believe these findings are nonetheless useful in describing the exposures identified and investigated by local public health authorities. In our study, investigators reported a known source and setting for 26% and 15% of domestic sporadic cases respectively, with percentages varying by pathogen. The percentage likely reflects the investigators’ confidence in identifying a source. Factors that likely influence this percentage include: the number of days from exposure to investigation and the associated recall bias, the investigators’ bias and knowledge of the sources as well as the transmission modes for the various pathogens, the case’s understanding or bias pertaining to their illness and its cause or source, and the effort made by the investigator. It should be recognized that with these limitations, the source is frequently unknown. Notably, the “known” sources of GI in our study are not systematically supported with information such as positive food samples or a statistical association through using case–control study methods, and are therefore highly susceptible to the various biases described above.

For the approximately one-quarter of cases with a known exposure source, we identified food as the primary exposure source for 54.2% of GI reported in Ontario from 2007 to 2009; food was also the most common exposure source for seven of the diseases examined: botulism, campylobacteriosis, cyclosporiasis, listeriosis, salmonellosis, VTEC-illness, and yersiniosis. In previous Ontario studies the overall proportion of GI attributed to food (when the source was known) was higher, ranging from 73.1% in 2003 to 75.9% in 2002 [5,6]. However, a more recent study in the Waterloo region of Ontario found the proportion attributed to food to be 57.3% between 1990 and 2004, and 41.2% between 2006 and 2010 [13]. Even if the percentage of foodborne transmission is indeed lower than previously identified, the magnitude is still large enough that continued vigilance of food along the entire “farm-to-fork” continuum is required.

Our finding that contact with animals was an important source of illness accounting for approximately 20% of all GI, was similar to that found by Dumoulin et al. for the years 2006 to 2010 (17.9%) [13]. This proportion was higher than that demonstrated in previous studies, where the proportion ranged from 1.0% in 2003 to 5.8% between 1997 and 2001 [5-7]. Further studies may be warranted to examine the cause of this increasing trend, as well as public health prevention strategies.

For amebiasis, hepatitis A, and shigellosis, person-to-person transmission was the most common primary exposure source. These findings reinforce the need for greater understanding of the importance of personal hygiene practices among cases, their household members, and other close contacts. In addition, more targeted educational information may be warranted for groups that are known to be at higher risk of acquiring these diseases, such as travellers to high-risk areas (e.g. Canadian residents visiting relatives abroad) and men who have sex with men.

The private home was the most common primary exposure setting for nine of the 14 diseases, accounting for less than one-half of all sporadic domestic GI cases. Food premises were identified as the risk setting for approximately one-third of these cases. Similar to the overall incidence rate calculations above, if the four parasitic diseases are removed from the risk setting proportions, private homes and food premises were identified as risk settings for 48.5% and 33.3% of cases, respectively. In previous years, the private home was also the most common overall risk setting for GI cases overall [5-7]. Moreover, when we examined only the cases exposed in the home, we found that food was still the most commonly reported source (62.6%). At 33.3%, the proportion of GI cases thought to have been acquired at a food premise appears to be increasing, as it was 14.1% between 1997 and 2001 [7], 15.0% in 2002 [6], and 20.7% in 2003 [5]. The importance of the home and food premises as risk settings for acquiring GI illness also emphasizes the continued need for a farm to fork approach to concurrently reducing pathogens in food as well as reducing risk at the consumer level.

### Case reporting

There were many factors that have had an impact on GI case reporting over the last decade, making comparisons over time difficult. Many of these changes may have increased the likelihood of case detection or reporting, improved case management, and ultimately, limited further transmission of disease. A full discussion of these factors is beyond the scope of this paper; however, in Ontario some of the factors included: changes in laboratory testing, changes in case definitions, and a change in the reporting system in 2005 from the Reportable Diseases Information System (RDIS) to iPHIS.

Reportable diseases represent the so-called ‘tip of the iceberg’; only a fraction of GI are reported to public health. While underreporting varies by pathogen, for every GI reported, there are an estimated 10–49 cases in the community that are not reported [14]. Underreporting is a well-known limitation of passive surveillance systems in general; however, since our data are population-based and likely representative, under-reporting will likely only differ slightly over time or for subpopulations, meaning the data can still be used to elucidate epidemiologic trends.

Overall, approximately one-third of cases were not successfully followed up. In public health practice, 100% follow-up is not attainable due to numerous factors, such as cases refusing to be interviewed or public health having incorrect contact information for the patient. Imperfect reporting is a common characteristic of passive surveillance systems such as the one in this study, where the cost of completely capturing data is likely prohibitive and must be balanced against timely capture of enough data to be useful (e.g., to detect outbreaks, vulnerable sub-populations, and trends) [7]. In general, our findings suggest that greater effort was made to follow up pathogens that were considered to have greater morbidity and mortality. Further interpretation of the differences in follow-up between diseases should be done with caution, as there are currently no unified and enforceable procedures for following up reportable diseases across all health jurisdictions in Ontario. Currently, the data collection requirements for jurisdictions are mandated by provincial regulations and infectious disease protocols, and there is no information regarding what a reasonably successful follow-up rate should be by disease.

## Conclusions

Reportable GI continue to be a burden in Ontario, with campylobacteriosis and salmonellosis remaining the most frequently reported illnesses. International travel is an important risk factor for most GI, with travel-related cases presenting distinct seasonal patterns from domestic cases. Obtaining good travel history from cases, and interpretation of other exposure and risk factor information is important for assessing the true burden of domestic disease, identifying clusters and trends, and targeting public health action. Most sources of infection for sporadic domestic GI are not identified; however, the most commonly reported suspect sources are food, animal contact and ill persons. While food premises continue to be a significant risk setting for foodborne GI, private homes remain the most commonly suspected setting for acquiring GI, underscoring continued need for public education on safe handling of food and animals, as well as proper hand hygiene practices.

## Competing interests

The authors declare that they have no competing interests.

## Authors’ contributions

DM and LV conceived of the study. LV, DM, KJ, and YW developed the study design and methodology. LV and KJ were involved with data management. LV conducted the data analyses and drafted the manuscript. LV, KJ, YW, and DM were involved with editing the manuscript and approved the final manuscript. All authors read and approved the final manuscript.

## Pre-publication history

The pre-publication history for this paper can be accessed here:

http://www.biomedcentral.com/1471-2458/12/970/prepub
